# Quantum probability theory as a common framework for reasoning and similarity

**DOI:** 10.3389/fpsyg.2014.00322

**Published:** 2014-04-11

**Authors:** Jennifer S. Trueblood, Emmanuel M. Pothos, Jerome R. Busemeyer

**Affiliations:** ^1^Department of Cognitive Sciences, University of CaliforniaIrvine, Irvine, CA, USA; ^2^Department of Psychology, City University LondonLondon, UK; ^3^Department of Psychological and Brain Sciences, Indiana UniversityBloomington, IN, USA

**Keywords:** quantum probability theory, conjunction fallacy, similarity judgment, decision-making, classical probability theory

The research traditions of memory, reasoning, and categorization have largely developed separately. This is especially true for reasoning and categorization, where the former has focused on logic and probability rules and the latter on similarity processes. For example, classical rules of logic are often considered the basis for human reasoning (Evans et al., 1991) in tasks such as the Wason selection task (Wason, 1966), which requires participants to use deductive reasoning to solve a logic puzzle involving four cards. Reasoning models are typically developed in terms of hypotheses for how relevant rules should be combined and applied to reach conclusions from the relevant premises (Braine et al., 1995). By contrast, in categorization, the predominant theoretical traditions (i.e., prototype theory and exemplar theory) have involved a similarity process (Wills and Pothos, 2012, provide an overview).

Such sharp distinctions between cognitive processes have started to break down. For example, Oaksford and Chater (1994) proposed a model of the Wason selection task based on information theory, rather than logical rules. Their model used information maximization in relation to uncertain hypotheses (cf. Anderson, 1991), which is an idea that could plausibly translate across other cognitive processes. Pothos (2010) showed how information maximization could apply to a learning task. Heit et al. (2012) argued that researchers should explore the connections between memory and reasoning. Tversky and Kahneman (1983), Shafir et al. (1990) proposed that when assessing the relative probability of statements about a hypothetical person, Linda, participants employ a process of similarity. Thus, the idea that similar or identical cognitive processes may underlie superficially disparate processes, like categorization and reasoning, is not new. What has been perhaps lacking is the development of specific models, which can be applied across different areas. Our purpose is to outline our ideas regarding such a model for probabilistic reasoning and similarity. We do so in the context of recent work with cognitive models based on quantum probability (QP) theory.

Many people are familiar with quantum mechanics. What is perhaps less known is that the ingenious physicists who developed quantum mechanics also invented a new theory of probability, since classical probability (CP) theory was inconsistent with their bold new theory of the physical world. QP theory refers to the rules for assigning probabilities to events from quantum mechanics, without the physics. QP theory is potentially applicable to any area where there is a need to compute probabilities. The motivation to adopt QP theory is typically informed by whether the empirical situation of interest reflects some key properties of QP theory, such as incompatibility, interference, superposition, and entanglement. For example, when two possibilities are incompatible, this means that it is impossible to concurrently assign a truth-value to both. So, if we are certain about one possibility, then we are necessarily uncertain about the other. In CP theory it is always possible to create a complete joint probability distribution for all available alternatives.

The quantum cognition research program is relatively new, but there have already been several notable applications, spanning conceptual combination (Aerts, 2009), perception (Atmanspacher et al., 2004), memory (Bruza et al., 2009), reasoning and decision-making (Busemeyer et al., 2011), and similarity (Pothos et al., 2013; for overviews see Busemeyer and Bruza, 2011 and Pothos and Busemeyer, 2013). We note that all these applications of QP theory in cognition have the form of standard cognitive models; they make claims regarding cognitive representations and processes, without any assumptions regarding the underlying neural substrate. In particular, quantum cognitive models do not require a quantum brain (the quantum brain hypothesis has been extremely controversial; Litt et al., 2006; Hammeroff, 2007).

QP theory is a geometric approach to probability where different possibilities (or events or questions) are represented as subspaces, of varying dimensionality, in a multidimensional Hilbert space. Hilbert spaces are like vector spaces, but with some additional properties. The system of interest (e.g., the cognitive state of a participant in an experimental task) is a vector in the Hilbert space, called the state vector. Probabilities are determined by projecting the state vector onto different subspaces and computing the squared length of this projection. For example, consider Tversky and Kahneman's (1983) famous Linda problem, where participants read a story about a hypothetical person, Linda, and were asked to judge the likelihood of different features of Linda. We assume that the mental state vector corresponds to the representation of the information about Linda, after reading the story. As illustrated in Figure 1, the relevant Hilbert space includes subspaces for all relevant features of Linda, such as whether she is a feminist. Then, to extract the probability that a participant in the experiment will consider Linda to be a feminist, we project the state vector onto the feminist subspace, and compute the squared length of this projection. Generally, probability assignment in QP theory essentially involves a process of overlap between the cognitive state (modeled by the state vector) and different possibilities (modeled by subspaces). Thus, probability assignment in QP theory is a plausible candidate for a similarity process as well. Indeed, Sloman (1993) independently presented a model of induction, based on ideas closely resembling the formal properties of probabilistic computation in QP theory.

**Figure 1:**
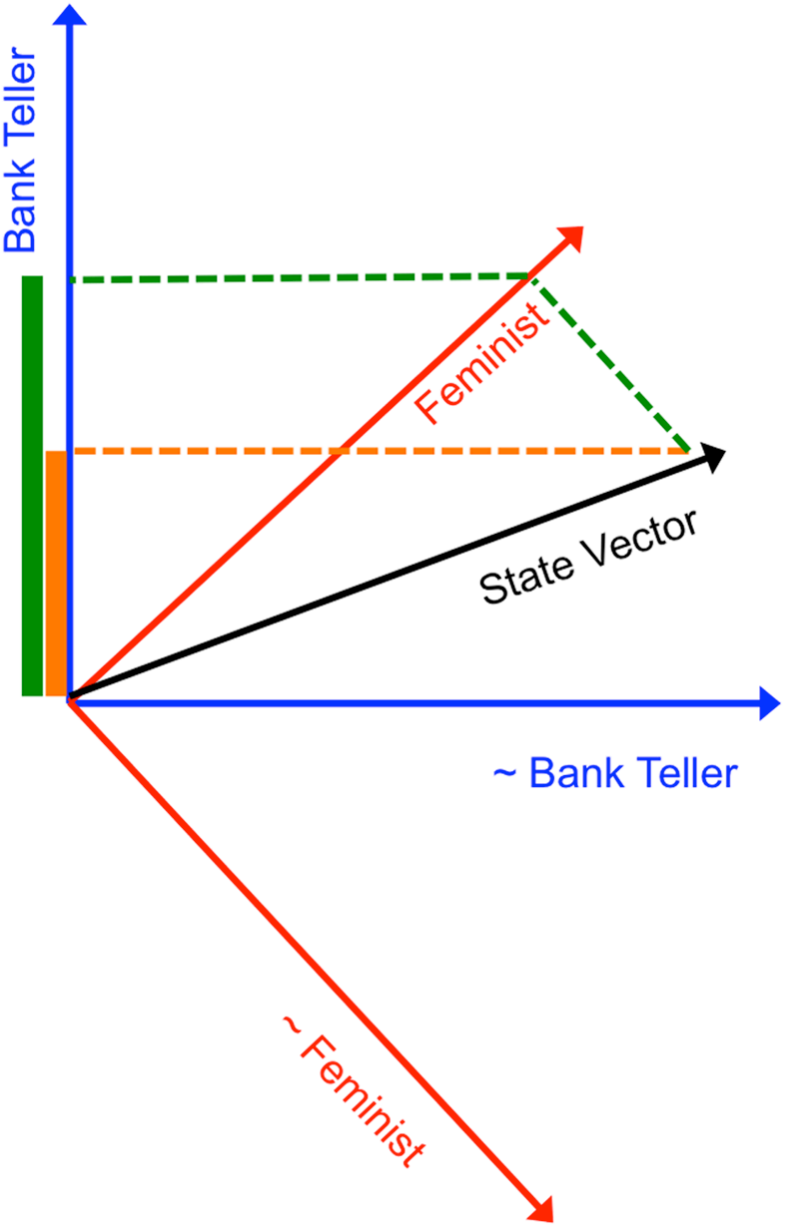
**The QP model for the conjunction fallacy**. To calculate the probability that Linda is a bank teller, we project the state vector (shown in black) onto the bank teller subspace (shown in blue) and compute the squared length of this projection (shown in orange). To calculate the conjunction that Linda is both a feminist and a bank teller, we first project the state vector onto the feminist subspace (shown in red) and then project it onto the bank teller subspace and compute the squared length of this projection (shown in green).

The process of projection in QP theory cognitive models is assumed to correspond to a process of thinking/evaluating the corresponding premise. However, for incompatible possibilities, it is impossible to identify a common projection to both relevant subspaces. This is equivalent to saying that it is impossible to concurrently assign a truth-value to incompatible observables. In order to compute a conjunction for incompatible observables (e.g., as needed for modeling the Linda experimental task), Busemeyer et al. (2011) postulated a process of sequential projection: the state vector is first projected onto the more likely predicate (feminism in the Linda example; cf. Gigerenzer and Goldstein, 1996) and then it is projected onto the less likely one (bank teller). The squared length of the resulting vector is the probability for the conjunction, according to QP theory. Note this number decomposes to the product of the probability of the marginal times the conditional, as one would expect from CP theory. Similar ideas of sequential projection have been used in other QP models to account for order effects in inference and causal reasoning (Trueblood and Busemeyer, 2011, 2012).

The basic computation Pothos et al. (2013) proposed for the quantum similarity model is nearly identical. Suppose we are modeling the similarity of object *A* to object *B*. Similarity assessment is assumed to involve thinking about the first object and then the second, a process which can be modeled by projecting first onto the *A* subspace and then onto the *B* one, and computing the squared length of the resulting vector. Thus, the computation for assessing the similarity between *A* and *B* is exactly the same as for computing the conjunction between *A* and *B*, with two exceptions. The first relates to the order of projection. In decision-making tasks, we assume that the task does not typically impose any constraints on which premise is considered first. A corresponding choice has to be made and choosing the more likely premise first is a reasonable assumption. In similarity tasks, the similarity question is often phrased in a way that imposes a particular directionality (Tversky, 1977) and it is this directionality which constraints the order of projection. The second exception relates to how the initial mental state vector is determined. In decision-making applications, the state vector is plausibly determined by the information initially presented. In similarity, the state vector is set so that it does not bias the similarity judgment toward either of the compared objects. One may question these assumptions. However, QP theory is a mathematical theory of probability and we cannot expect psychological predictions to emerge without introducing some psychological assumptions too. The assumptions regarding projection order are exactly of this kind. It is worth noting that the quantum model for the conjunction fallacy predicts that if a person judges an unlikely event U before judging the conjunction of the unlikely event U and a likely event L, the effect is smaller than when the questions are in the opposite order, which has been confirmed experimentally by Stolarz-Fantino et al. (2003) and Gavanski and Roskos-Ewoldsen (1991).

Overall, the decision-making model for the conjunction fallacy (Busemeyer et al., 2011) and the similarity model for Tversky's (1977) findings have similar forms. We think that this is a novel accomplishment, in that we provide a formal and testable expression of the idea that similarity and reasoning processes may involve the same or very similar cognitive mechanisms. Of course, the value of the modeling lies in the extent to which it can inform empirical prediction. Though speculative, we currently think there are a number of promising directions. For example, order effects in similarity judgments can arise because of differences in the degree of knowledge we have for one of the compared elements vs. the other (such differences can be related to the relative dimensionality of the corresponding subspaces). It is likewise possible that, in decision-making tasks, conjunctions in certain directions will be more natural than others. Tversky (1977) also reported a diagnosticity effect; where the similarity between two options resulted in a third, separate option being preferred in a matching task. Participants in this task were asked to decide which country was most similar to Austria. In one condition, the candidate choices were Sweden, Hungary, and Poland, and participants favored Sweden. However, when the candidate choices where changed to Sweden, Norway, and Hungary, participants preferred Hungary. Tversky explained the effect through a change in grouping–Eastern vs. Western Europe in the first condition and Nordic vs. non-Nordic in the second. This diagnosticity effect is analogous to a similarity effect in decision-making: when introducing an option similar to one of the existing options, this decreases the desirability of the similar options (cf. Trueblood et al., 2013). The quantum similarity model can capture the diagnosticity effect and the same mechanism has the potential to accommodate the corresponding result in decision-making.

Likewise, the order of projections in the quantum similarity model allows for violations of the triangle inequality in similarity judgments and perhaps similar violations in decision-making can arise in the same way. The triangle inequality is a fundamental property of distance that must be obeyed by similarity measures based on simple functions of distance. In similarity judgments, the triangle inequality can be expressed as *Similarity(A, B) > Similarity(A, C) + Similarity(C, B)*. Tversky (1977) reported a violation of the triangle inequality by having participants judge the similarity between three countries: Russia, Jamaica, and Cuba. He found that the similarity of Russia and Jamaica was smaller than the similarity of Russia and Cuba plus the similarity of Cuba and Jamaica.

Is it meaningful to consider whether a general model of cognition could be based on QP principles? This is certainly an exciting proposition for researchers in the quantum cognition area. But a few qualifications need be noted. First, CP theory cognitive models can be and have been extremely successful too (e.g., Griffiths et al., 2010). A preference for the unique features of QP theory, against CP theory, can be motivated in situations where there is evidence of context effects, interference effects, and sequence effects. It is also possible that there will be empirical findings beyond both CP theory and QP theory. In fact, QP theory is a highly constrained framework and we already have simple, non-parametric tests, which can test for consistency with QP principles (Wang and Busemeyer, 2013).

All of these possibilities provide exciting directions for future research. Although we did not discuss memory processes explicitly, there has been initial work in developing a QP model of memory recognition (Busemeyer and Trueblood, 2010). In sum, we think that the current quantum cognitive models provide an encouraging starting point in exploring the commonalities between memory, reasoning, and categorization, because of the simplicity of the basic ideas and their applicability across areas.

## Conflict of interest statement

The authors declare that the research was conducted in the absence of any commercial or financial relationships that could be construed as a potential conflict of interest.
